# Synergistic effects of plant growth regulators, riboflavin, and iron nanoparticles on secondary metabolites in micropropagated *Ceratonia siliqua L.*

**DOI:** 10.1038/s41598-026-55883-1

**Published:** 2026-06-04

**Authors:** Lobna S. Taha, Nora M. Youssef, Eman A. Ibrahim, Iman M. El-Sayed

**Affiliations:** 1 Ornamental Plants Woody Trees Department, Agriculture and Biological Research Institute, National Research Centre (NRC), 33 El Buhouth St, Dokki, Giza, 12622 Egypt; 2 Plant Biochemistry Department, Agriculture and Biological Research Institute, National Research Centre (NRC), 33 El Buhouth St, Dokki, Giza, 12622 Egypt

**Keywords:** *Ceratonia siliqua*, Micropropagation, Riboflavin, Auxins, Fe nanoparticles, Secondary metabolites, HPLC, Plant sciences, Plant development, Secondary metabolism

## Abstract

Plant growth regulators (PGRs) play a crucial role in regulating the biochemical and physiological processes. Recently, nanoparticle-based approaches have attracted increasing attention for improving in vitro rooting efficiency. *Ceratonia siliqua* L. is a significant tree species utilized for landscaping and medicinal purposes; however, its conventional propagation is limited, making micropropagation a viable alternative. This study aimed to optimize in vitro multiplication, rooting, and secondary metabolite accumulation of *C. siliqua* through the use of different PGR combinations, Murashige and Skoog (MS) medium strength, and riboflavin during the multiplication stage, while evaluating the effect of iron oxide nanoparticles (Fe₂O₃ NPs) exclusively during the rooting stage. During establishment and multiplication, explants were cultured on full- or half-strength MS media supplemented with riboflavin in combination with benzyladenine (BA), naphthaleneacetic acid (NAA), and gibberellic acid (GA₃). Rooting experiments were conducted using indole-3-butyric acid (IBA; 2, 3, and 4 mg L^− 1^) applied alone or in combination with Fe₂O₃ NPs (5 and 10 mg L^− 1^). Chemical analyses were performed only on responsive multiplication treatments and compared with the mother plant. The highest shoot number (3.33 shoots per explant), shoot length (46.33 mm), and leaf number (42.67 leaves per explant) were recorded on full-strength MS medium supplemented with 1 mg L^− 1^ riboflavin, 1 mg L^− 1^ BA, 0.1 mg L^− 1^ NAA, and 0.1 mg L^− 1^ GA₃, which also resulted in the highest flavonoid content in the multiplied shootlets. For rooting, the highest rooting percentage and root number were obtained with 3 mg L^− 1^ IBA alone or in combination with 5 mg L^− 1^ Fe₂O₃ NPs. Regarding secondary metabolites, shoots on ½ MS with 1 mg L⁻¹ riboflavin and 0.5 mg L⁻¹ BA yielded high phenolic content, nearly matching the mother plant, while significantly surpassing it in DPPH antioxidant activity. HPLC confirmed this enhanced bioactivity via the unique accumulation of gallic acid, rutin, and vanillin.

**Supplementary Information:**

The online version contains supplementary material available at 10.1038/s41598-026-55883-1.

## Introduction

Nanoparticles (NPs) can be widely used in agriculture, industry, consumer, and medicine^1–3^. Iron is an essential mineral for the metabolism of plants. It also works as a cofactor for different enzymes directly or indirectly involved in synthesizing DNA and respiration. Moreover, it also acts as a cofactor for various enzymes involved in the redox reaction, such as hormone synthesis, respiration, and photosynthesis^4–6^. Iron nanoparticles are widely utilized because they are less expensive than other non-toxic metallic nanoparticles and have properties like biocompatibility and solubility in water^7,8^.

Micropropagation is a biotechnological technique to obtain plant tissues, cells, or organs in synthetic nutritional media under aseptic and controlled environments. It is suitable for solving some propagation problems, such as difficult propagation and the rooting of trees by traditional methods^9–11^. Additionally, large-scale production, pathogen-free plant preparation, and preserving endangered species stocks^12–14^. Plant growth regulators, or PGRs, are important regulatory molecules that impact plant development. They affect plant physiology, including shootlet proliferation, root elongation, seed germination, and bud dormancy^15–17^. The effect of using auxin (IBA) alone or with iron nanoparticles to improve rooting was studied^18–20^.

*Ceratonia siliqua* L., tree, called Carob, belongs to the family Leguminosae or (Fabaceae) and is commonly cultivated in the Mediterranean^16^ for its ornamental, landscaping, windbreaks, and forestation as well as nutritional and medicinal value^21^. Carob tree tolerates adverse conditions, alkali soils, and drought, and is highly tolerant to heat and dry atmospheres. Hence, it has been used naturally for land reclamation and decreased soil erosion in most Mediterranean areas^22^. Carob pods are rich sources of polyphenols such as ellagic acid, Gallic acid, flavonoids, and anthocyanins. Moreover, carob pulp has benefits in food with high total sugar content (48 56%). Additionally, Carob contains approximately 18% cellulose and hemicellulose, as well as several minerals, including P, Ca, Mg, Na, Fe, Cu, and Zn. Additionally, carob extracts have many beneficial impacts on health. For instance, cholesterol reduces activity in people suffering from hypercholesterolemia^23,24^. Additionally, the propagation methods of Carob could be by way of seeds, budding, cutting, or grafting^25^, but this species is one of the most difficult species to form roots, and its seeds are hard to germinate because the coat of the seed is tough and cannot absorb water well^26^. Like other woody trees, Carob is hard to propagate using tissue culture, and only some studies have been successful. There is no information available about the impact of nanomaterials on enhanced growth rate and root stimulation in *Ceratonia siliqua* L, although some studies have focused on the role of plant growth regulators^24,27,28^.

This study aims to develop an efficient method for shortening the production cycle of *Ceratonia siliqua* L., which is common among vigorous, ornamental, and medicinal trees that do not root easily. Therefore, many factors known to be paramount in micropropagation were tested to overcome these problems. In addition, the effect of these factors on the content of secondary metabolism compared to the mother plant was studied.

## Materials and methods

This experiment was carried out during (2023–2024) on *Ceratonia siliqua L.*, at the Tissue Culture Laboratory, Central Laboratories, Department of Ornamental Plants and Woody Trees, National Research Centre (NRC), Egypt.

### Plant source

Samples of *Ceratonia siliqua* L. (nodal stem) were collected from a female tree planted at the Horticulture Research Institute (HRI), Agricultural Research Centre, Giza, Egypt, in March 2023. The plant material used in this study was identified by agricultural engineer Traez Labib at the Orman Garden Herbarium, Giza. A voucher specimen (No. M281) was deposited in the National Research Centre Herbarium (CAIRC) by Professor Mona Marzouk of the Phytochemistry and Plant Systematics Department.

### Plant material and sterilization

The freshly cut nodal stems of *Ceratonia siliqua* L. were cleaned with soap for 30 min, then washed with running tap water for 1 h. The rinsed explants were immersed in 70% ethanol for 20 s., then treated with 15% sodium hypochlorite (Clorox) containing 0.01% Tween 20 for 10 min, and finally washed with sterile water 4 times. The explants were then sterilized in 0.1% HgCl2 solution for 7 min. Finally, explants were washed with four changes of sterilized distilled water under aseptic conditions. The Occupational Safety and Health Committee’s guidelines were followed when using HgCl_2_.

### Culture medium

Explants were cultured on full-strength MS medium, supplemented with 25 g L^− 1^ sucrose and 8 g L^− 1^ agar. The pH of the medium was adjusted to 5.7 ± 0.2 using HCl or NaOH, and the medium was then autoclaved at 121 °C for 15 min and 1.2 kg cm⁻² for sterilization.

### Culture incubation

The in vitro cultures were kept at 24 ± 1 °C in a growth chamber with fluorescent light that provided 3k lux of light over 16 h of photoperiods.

### **Experiment treatments**

The obtained proliferated shootlets on MS basal medium were subcultured after two months to serve the two main experiments:

#### The first experiment for the multiplication treatments is as follows

In the multiplication stage, riboflavin was added to reduce phenolic oxidation, promote shoot development, or enhance secondary metabolite accumulation.

**Table:** 

**T1**: ½ MS + 1.0 mg L^− 1^ riboflavin	**T6**: full MS + 1.0 mg L^− 1^ riboflavin
**T2**: ½ MS + 1.0 mg L^− 1^ riboflavin + 0.5 mg L^− 1^ BA	**T7**: full MS + 1.0 mg L^− 1^ riboflavin + 0.5 mg L^− 1^ BA
**T3**: ½MS + 1.0 mg L^− 1^ riboflavin + 1.0 mg L^− 1^ BA	**T8**: full MS + 1.0 mg L^− 1^ riboflavin + 1.0 mg L^− 1^ BA
**T4**: ½ MS + 1.0 mg L^− 1^ riboflavin + 0.5 mg L^− 1^ BA + 0.1 mg L^− 1^ NAA + 0.1mg L^− 1^ GA_3_	**T9**: full MS + 1.0 mg L^− 1^ riboflavin + 0.5 mg L^− 1^ BA + 0.1 mg L^− 1^ NAA + 0.1 mg L^− 1^ GA_3_
**T5**: ½ MS + 1.0 mg L^− 1^ riboflavin + 1.0 mg L^− 1^ BA + 0.1 mg L^− 1^ NAA + 0.1 mg L^− 1^ GA_3_	**T10**: full MS + 1.0 mg L^− 1^ riboflavin + 1.0 mg L^− 1^ BA + 0.1 mg/L NAA + 0.1mg L^− 1^ GA_3_

Using a randomized complete design (RCD) with three replicates of jars, and each jar contains three explants for each treatment. The number and length of shootlets per explant, and the number of leaves for each treatment, were determined following the three-month incubation period.

#### The second experiment for optimizing shooting and rooting abilities

IBA at different levels (2, 3, and 4 mg L^− 1^) and dispersed Fe oxide NPs (5 and 10 mg L-1 each), as described by Zafar et al.^29^, were used alone or in combination with them in the MS culture medium. The investigation employed specific Fe NPs obtained from Sigma Co., USA. The NRC Electron Microscope unit was utilized to characterize the Fe NPs. IBA and Nano-Ferric Oxide (Fe_2_O_3_ NPs) were supplemented with varied treatments as follows:

**Table:** 

**T1**: Control (A)	**T7**: A + 3.0 mg L^− 1^ IBA
**T2**: A + 5.0 mg L^− 1^ Fe_2_O_3_ NPs	**T8**: A + 3.0 mg L^− 1^ IBA + 5 mg L^**− 1**^ Fe_2_O_3_ NPs
**T3**: A + 10.0 mg L^− 1^ Fe_2_O_3_ NPs	**T9**: A + 3.0 mg L^− 1^ IBA + 10 mg L^**− 1**^ Fe_2_O_3_ NPs
**T4**: A + 2.0 mg L^− 1^ IBA	**T10**: A + 4.0 mg L^− 1^ IBA
**T5**: A + 2.0 mg L^− 1^ IBA + 5.0 mg L^**− 1**^ Fe_2_O_3_ NPs	**T11**: A + 4.0 mg L^− 1^ IBA + 5 mg L^**− 1**^ Fe_2_O_3_ NPs
**T6**: A + 2.0 mg L^− 1^ IBA + 10.0 mg L^**− 1**^ Fe_2_O_3_ NPs	**T12**: A + 4.0 mg L^− 1^ IBA + 10 mg L^**− 1**^ Fe_2_O_3_ NPs

Control (A): ½ MS + 1 mg L^− 1^ riboflavin + 0.1 mg/L GA_3_ + 2 g L^− 1^ charcoal)

**Fig. 1:**
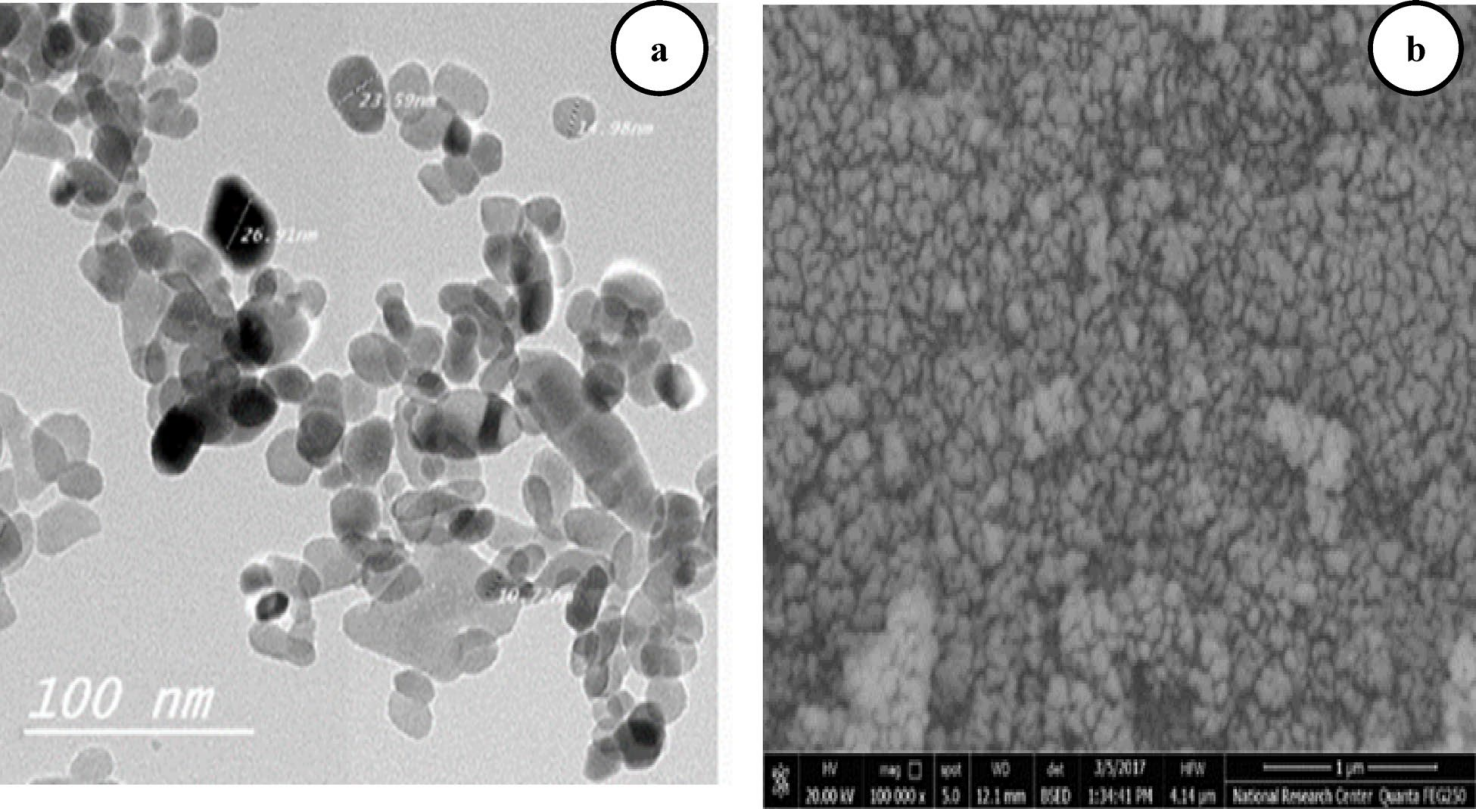
Scanning electron microscopy image of Iron oxide NPs, (**a**) at 100 nm, (**b**) at 12 nm.

The specification of the Fe_2_O_3_ NPs in the present experiment is indicated in Table 1; Fig. 1.

**Table 1:** Iron Oxide NP specification.

Nanoparticle	Phase (XRD)	Particle size (TEM)	Particle size (TEM)	Surface area (BET, *P*/Po ≤ 0.35)	Purity (EDX, wt% / at%)
Nano-Hematite (Fe₂O₃)	hematite	˂50 nm	˂50 m^2^g^− 1^	˃50 m^2^g^− 1^	O:23.06 wt%, 51.13 at%Fe: 76.94wt%, 48.87at%

#### Shooting and rooting behaviour

The number of shootlets per explant, shootlet length (mm), number of leaves, root percentage (%), number of roots per shootlet, and root length (mm) were recorded for each treatment after three months.

#### Acclimatization

After two months, the height of the shoots, as well as the quantity and length of roots per generated shoot, were noted. After being removed from the rooting culture medium, some of the rooted plantlets were cleaned and put in plastic pots with a 1:1:1 mixture of clay, peat, and perlite. Recently potted plantlets were covered with polythene bags for three weeks before being moved into the greenhouse. A fungicide was applied to the soil. During acclimatization, we removed rooted plantlets from the planting jars, submerged them in a fungicide, and then cleaned the roots with a fungicide-containing solution. Each plant must be covered to help it become acclimated to the outdoors. To ensure the plantlets adapt effectively to their new surroundings, a variety of treatments are employed, including irrigation tailored to the plant’s specific requirements and fertilisation with N-P-K 10-10-10, five to seven days later.

### Chemical analysis

The shootlets were chemically analyzed after the multiplication stage.

**Sample preparation for chemical composition analysis**.

Fresh samples of shootlets *Ceratonia siliqua* were used for chemical composition analysis. Approximately 0.1 g of fresh weight (FW) was accurately weighed and homogenized with 5 mL of analytical-grade ethanol to facilitate the extraction of soluble constituents. The homogenate was subsequently filtered through Whatman No. 1 filter paper to obtain a clear ethanolic extract. The resulting filtrate was collected and stored under suitable conditions until its chemical composition could be analysed.

#### Total phenolic content

Total phenolic content (TPC) of the ethanol extracts was determined using the Folin–Ciocalteu colorimetric method with gallic acid as a standard. Serial concentrations of gallic acid (1–10 µg mL⁻¹) were prepared to construct the calibration curve. The reaction mixture consisted of 2.5 mL of 10% (v/v) Folin–Ciocalteu reagent, 2.5 mL of ethanol extract, and 2.5 mL of 7.5% sodium carbonate solution. The mixtures were incubated at 45 °C for 45 min to allow color development. After incubation, absorbance was measured at 760 nm using a spectrophotometer against a reagent blank. Total phenolic content was calculated from the gallic acid calibration curve and expressed as µg gallic acid equivalents per gram fresh weight (µg GAE g⁻¹ FW). All measurements were performed in triplicate to ensure reproducibility^30^.

#### Total flavonoid content

Quercetin was utilized as a reference in the study to calculate the total flavonoid concentration using the aluminium chloride method. 0.3 ml of 5% NaNO2 was added to the extracts or standard solutions (quercetin, 1–12 µg mL^− 1^). Following a 5-minute incubation period, 0.3 mL of 10% AlCl_3_ was added. This was followed by the addition of 2 mL of NaOH (1 mol L⁻¹) and a 6-minute incubation. After the solutions were well-mixed, the absorbance at 510 nm was measured using a spectrophotometer against a prepared reagent blank^31^.

#### Total tannin content

Total tannin content (TTC) was determined using the Folin–Ciocalteu colorimetric method with tannic acid as a standard. Briefly, 7.5 mL of distilled water was mixed with the ethanol extract or standard tannic acid solutions (20–120 µg mL⁻¹). Subsequently, 0.5 mL of Folin–Ciocalteu reagent was added, followed by 1 mL of sodium carbonate solution (35%, w/v). The reaction mixture was adjusted to a final volume of 10 mL with distilled water and allowed to develop under ambient conditions. Absorbance was measured at 775 nm using a spectrophotometer (Unico UV-2000), with a reagent blank prepared under the same conditions. Total tannin content was calculated from the calibration curve of tannic acid and expressed as µg tannic acid equivalent per gram fresh weight (µg TAE g⁻¹ FW). All measurements were performed in triplicate to ensure accuracy and reproducibility^32^.

#### Total sugar content

Total sugar content was determined using the colorimetric method described by Dubois et al.^33^. One milliliter of the extract was mixed with 1 mL of 5% phenol solution, followed by the rapid addition of 5 mL of concentrated sulfuric acid. The mixture was thoroughly vortexed and allowed to stand at room temperature for 30 min to develop colour. Absorbance was measured at 490 nm using a UV–Vis spectrophotometer. Total sugar content was quantified using a glucose calibration curve and expressed as mg glucose equivalents per gram of fresh weight.

#### The HPLC conditions for the identification of the extracted compounds

The phenolic profile of *Ceratonia siliqua* extract was analyzed using a high-performance liquid chromatography (HPLC) system (Agilent 1260, Agilent Technologies, USA) equipped with an Eclipse C18 column (4.6 × 250 mm, 5 μm particle size). Before injection, sample extracts and phenolic standards were filtered, and 5 µL of each solution was injected into the HPLC system. The mobile phase consisted of water (solvent A) and acetonitrile containing 0.05% trifluoroacetic acid (solvent B), delivered at a flow rate of 0.9 mL min⁻¹. The column temperature was maintained at 40 °C. Separation was achieved using a linear gradient program as follows: 82% A at 0 min; 82% A from 0 to 1 min; 75% A from 1 to 11 min; 60% A from 11 to 18 min; followed by re-equilibration to 82% A from 18 to 24 min. Detection was performed at 280 nm.

#### Antioxidant Activity (DPPH)

The free radical–scavenging potential of *Ceratonia siliqua* ethanol extracts was assessed using the DPPH (2,2-diphenyl-1-picrylhydrazyl) assay, following the methodology of Ye et al.^34^ with slight modifications. Extracts were prepared at a concentration of 100 µg mL^− 1^ and mixed with 3.0 mL of 0.1 mM DPPH solution in ethanol. The reaction mixtures were incubated in the dark for 30 min, and the absorbance was subsequently recorded at 517 nm to evaluate the radical-scavenging activity.

#### In vitro total antioxidant capacity assay

The total antioxidant capacity of *Ceratonia siliqua* extracts was determined using the phosphomolybdenum assay. In brief, 20 mL of distilled water was combined with 1 mL of 0.6 M sulfuric acid, 28 mM sodium phosphate, and 4 mM ammonium molybdate, and the final volume was adjusted to 50 mL with additional distilled water. For the assay, 3 mL of this reagent solution was transferred to each test tube, followed by the addition of 0.3 mL of extract at different concentrations, including 100 µg mL^− 1^. The tubes were incubated at 95 °C for 90 min. After cooling to room temperature, the absorbance of each solution was measured at 695 nm using a UV–visible spectrophotometer, with a blank containing ethanol and reagent used as a reference^35^.

### Statistical analysis

COSTATV-63 Duncan^36^, was used to evaluate the data, and a randomized complete design with three replicas for each treatment was used. Using new multiple-range tests at *p* ≤ 0.05, a one-way analysis of variance (ANOVA) was used to establish the significance.

## Results

### Establishment stage

**Fig. 2:**
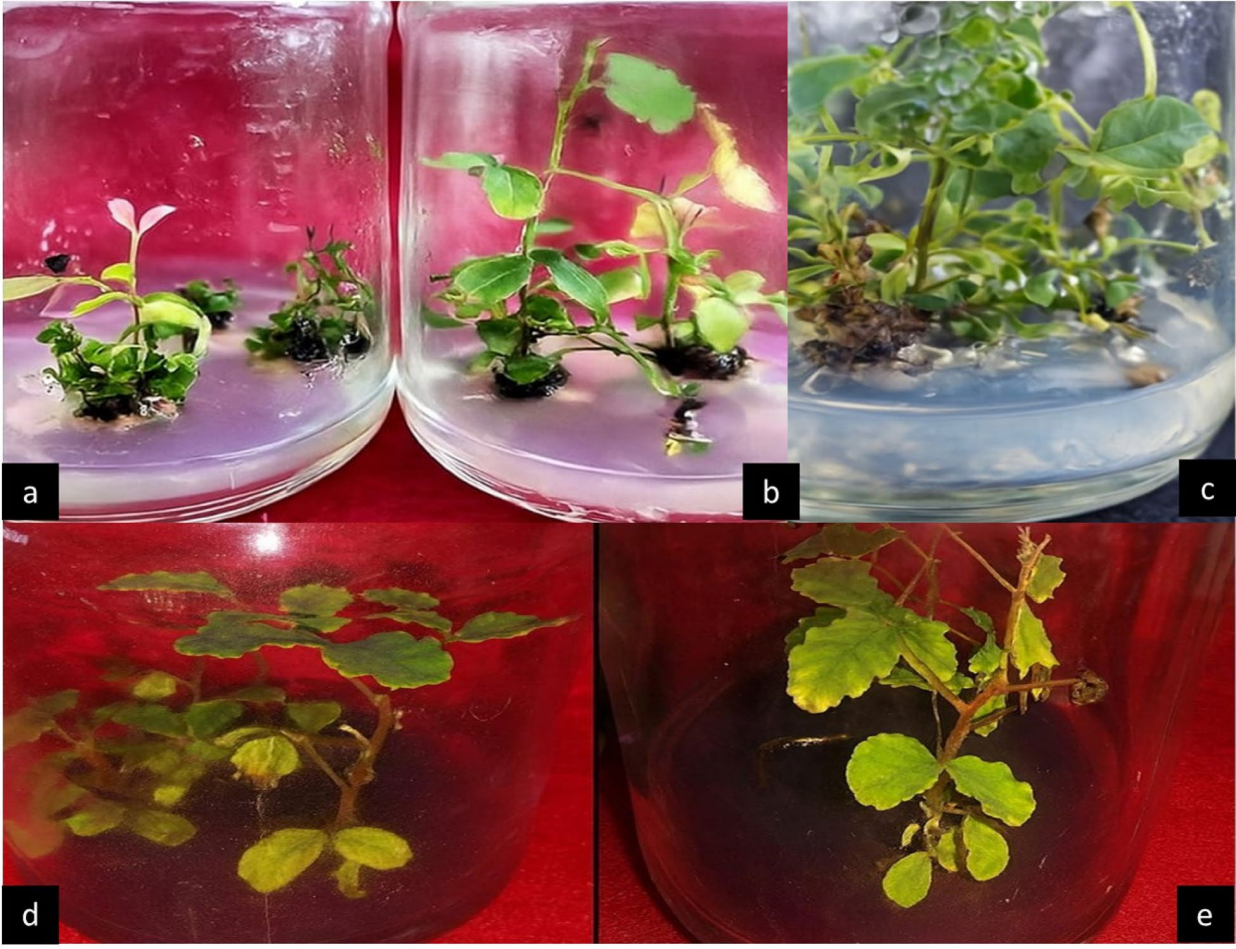
Micropropagation of *Ceratonia siliqua* shooting behavior; (**a**) Shoot proliferation in vitro with application of ½ MS + 1 mg L^-1^ Ribo, (**b**) applying with ½ MS + 1 mg L^-1^ Ribo + 0.5 mg L^-1^BA, (**c**) treating culture media with Full MS + 1 mg L^-1^ Ribo + 1 mg/L BA + 0.1 mg L^-1^ NAA + 0.1 mg L^-1^ GA_3_, **(d**,** e**) Shoot proliferation in vitro with application of 3 mg L^-1^ IBA + 5 mg L^-1^ Fe_2_O_3_NPs.

Table 2 shows that shootlet explants recorded 100% survival in most treatments, except those cultured on full-strength MS medium supplemented with 1 mg L⁻¹ riboflavin alone or in combination with BA at 0.5 or 1.0 mg L⁻¹. The highest mean shoot number per explant, shoot length, and number of leaves per shootlet were recorded on full-strength MS medium supplemented with 1 mg L⁻¹ riboflavin, 1 mg L⁻¹ BA, 0.1 mg L⁻¹ NAA, and 0.1 mg L⁻¹ GA₃, producing 3.33 shoots per explant, 46.33 mm shoot length, and 42.67 leaves per shootlet, respectively. This was followed by half-strength MS medium supplemented with the same additives, which produced 2.67 shoots per explant, 30.33 mm shoot length, and 31.67 leaves per shootlet. The lowest values for shoot number, shoot length, and leaf number (1.33 shoots, 19.33 mm, and 12.67 leaves per shootlet, respectively) were observed in explants cultured on full-strength MS medium supplemented with 1 mg L⁻¹ riboflavin alone (Fig. 2).

**Table 2:** Shooting ability of *Ceratonia siliqua* L. is affected by different concentrations of Ribo, BA, NAA, and GA_3_.

Treatments	Survival	Shoot number/explant	Shoot length (mm)	Leaves number/explant
½ MS + 1 mg L^− 1^ Ribo	100 ± 0.00^a^	1.33 ± 0.51^b^	21.33 ± 1.53^cd^	13.33 ± 1.53^f^
½ MS + 1 mg L^− 1^ Ribo + 0.5 mg L^− 1^ BA	100 ± 0.00^a^	2.33 ± 0.51^ab^	21.67 ± 2.08^cd^	20.67 ± 2.52^de^
½ MS + 1 mg L^− 1^ Ribo + 1 mg L ^−1^BA	100 ± 0.00^a^	2.67 ± 1.00^ab^	23.67 ± 1.15^cd^	22.33 ± 1.53^cd^
½ MS + 1 mg L^− 1^ Ribo + 0.5 mg L^− 1^ BA + 0.1 mg L^− 1^ NAA + 0.1 mg L^− 1^ GA_3_	100 ± 0.00^a^	2.33 ± 0.50^ab^	23.00 ± 2.00^cd^	24.33 ± 2.31^c^
½ MS + 1 mg L^− 1^ Ribo + 1 mg L^− 1^ BA + 0.1 mg L^− 1^ NAA + 0.1 mg L^− 1^ GA_3_	100 ± 0.00^a^	2.67 ± 0.51^ab^	30.33 ± 2.08^b^	31.67 ± 1.53^b^
Full MS + 1 mg L^− 1^ Ribo.	80.00 ± 3.00^c^	1.33 ± 0.58^b^	19.33 ± 2.52^d^	12.67 ± 1.53^f^
Full MS + 1 mg L^− 1^ Ribo + 0.5 mg L^− 1^ BA	83.00 ± 2.00^b^	1.67 ± 1.53^b^	20.33 ± 2.08^d^	15.00 ± 2.00^f^
Full MS + 1 mg L^− 1^ Ribo + 1 mg L^− 1^ BA	88.0 ± 2.00^b^	2.00 ± 1.00^ab^	21.33 ± 2.08^cd^	19.00 ± 2.00^e^
Full MS + 1 mg L^− 1^ Ribo + 0.5 mg L^− 1^ BA + 0.1 mg L^− 1^ NAA + 0.1 mg L^− 1^ GA_3_	100 ± 0.00^a^	2.33 ± 0.58^ab^	25.33 ± 1.53^c^	23.33 ± 1.53^c^
Full MS + 1 mg L^− 1^ Ribo + 1 mg L^− 1^ BA + 0.1 mg L^− 1^ NAA + 0.1 mg L^− 1^ GA_3_	100 ± 0.00^a^	3.33 ± 0.58^a^	46.33 ± 1.53^a^	42.67 ± 1.15^a^

Data are presented as mean ± standard deviation (SD) based on three replicates (*n* = 3). Means within the same column followed by identical letter (s) are not significantly different, as determined by Duncan’s multiple range test at a significance level of *p ≤* 0.05. MS = Murashige and Skoog medium; Ribo = riboflavin; BA = 6-benzyladenine; GA_3_= gibberellic acid; NAA = naphthalene acetic acid.

### Micropropagation

Shoot proliferation and rooting ability of *Ceratonia siliqua* were evaluated under different concentrations of Fe₂O₃ nanoparticles (5 and 10 mg L⁻¹) and IBA (2, 3, and 4 mg L⁻¹), applied individually or in combination. Data in Table 3 indicated that the highest shoot number, shoot length, and leaf number were obtained from shootlets cultured on MS medium supplemented with 2 mg L⁻¹ IBA + 10 mg L⁻¹ Fe₂O₃ NPs, producing 2.5 shoots, 46.0 mm shoot length, and 13.00 leaves per explant, respectively. This was followed by shootlets cultured on MS medium supplemented with 3 mg L⁻¹ IBA alone, producing 2.4 shoots, 41.7 mm shoot length, and 12.33 leaves per shootlet. Regarding rooting, shootlets cultured on half-strength MS medium supplemented with 3 mg L⁻¹ IBA alone or in combination with 5 mg L⁻¹ Fe₂O₃ NPs showed the highest rooting percentage and root number (50.0% rooting with 2.02 roots, and 50.0% rooting with 2.30 roots, respectively). The longest roots (120 mm) were obtained from shootlets cultured on MS medium supplemented with 3 mg L⁻¹ IBA and 5 mg L⁻¹ Fe₂O₃ NPs (Fig. 2).

**Table 3:** Micropropagation of *Ceratonia siliqua* L., under **the** effect of Fe NPs and IBA at various concentrations.

Treatments	Shoot number/explant	Shoot length (mm) explant	Leaves number/explant	Rooting (%)	Roots number/explant	Root length (mm)/explant
Control (A)	1.80 ± 0.25^ce^	45.00 ± 1.00^a^	12.0 ± 1.00^ab^	0.00 ± 0.00^c^	0.00 ± 0.00^c^	0.00 ± 0.00^g^
Control + 5 mg L^− 1^ Fe_2_O_3_ NPs	1.4 ± 0.20^ef^	38.3 ± 1.53^bd^	12.30 ± 0.58ab	33.30 ± 2.89^c^	1.00 ± 0.20^b^	65.00 ± 5.00^d^
Control + 10 mg L^− 1^ Fe_2_O_3_ NPs	1.9 ± 0.23^be^	37.3 ± 2.52^bd^	8.30 ± 0.58^e^	16.70 ± 2.89^d^	1.00 ± 0.20^b^	86.66 ± 2.89^cd^
Control + 2 mg L^− 1^ IBA	1.9 ± 0.17^be^	26.3 ± 2.52^e^	13.00 ± 1.00^a^	0.00 ± 0.00^e^	0.00 ± 0.00^c^	0.00 ± 0.00^g^
Control + 2 mg L^− 1^ IBA + 5 mg L^− 1^ Fe_2_O_3_ NPs	2.3 ± 0.20^ab^	37.3 ± 2.52^bd^	11.66 ± 1.15^ab^	16.70 ± 1.150^d^	1.00 ± 0.20^b^	105.0 ± 1.73^b^
Control + 2 mg L^− 1^ IBA + 10 mg L^− 1^ Fe_2_O_3_ NPs	2.50 ± 0.30^a^	46.00 ± 1.00^a^	13.00 ± 1.0 ^a^	0.0 ± 0.00^e^	0.00 ± 0.00^c^	0.00 ± 0.00^g^
Control + 3 mg L^− 1^ IBA	2.40 ± 0.20^a^	41.70 ± 2.08^ab^	12.33 ± 1.53^ab^	50.0 ± 5. 00^a^	2.02 ± 0.58^a^	86.70 ± 3.51^cd^
Control + 3 mg L^− 1^ IBA + 5 mg L^− 1^ Fe_2_O_3_ NPs	1.50 ± 0.23^df^	39.30 ± 1.15^bc^	9.30 ± 0.58^de^	0.00 ± 5.00^a^ 50	2.30 ± 0.58^a^	120.00 ± 2.00^a^
Control + 3 mg L^− 1^ IBA + 10 mg L^− 1^ Fe_2_O_3_ NPs	1.10 ± 0.17^f^	36.30 ± 1.15^cd^	11.00 ± 1.0b^c^	16.30 ± 1.53^c^	1.16 ± 0.30^b^	90.00 ± 2.89^c^
Control + 4 mg L^− 1^ IBA	1.70 ± 1.00^ce^	36.30 ± 2.08^cd^	12.33 ± 1.15^ab^	50.00 ± 5.00^a^	1.10 ± 0.20^b^	21.70 ± 2.00^f^
Control + 4 mg L^− 1^ IBA + 5 mg L^− 1^ Fe_2_O_3_ NPs	2.00 ± 1.00^ad^	33.30 ± 2.89^d^	10.00 ± 1.00^cd^	38.33 ± 9.62^b^	1.100 ± 0.20^b^	60.00 ± 2.00^e^
Control + 4 mg L^− 1^ IBA + 10 mg L^− 1^ Fe_2_O_3_ NPs	2.00 ± 1.00^ad^	35.00 ± 2.00^cd^	8.00 ± 1.00^e^	0.00 ± 0.00^e^	0.0 ± 0.00^c^	0.00 ± 0.00^g^

Data are presented as mean ± standard deviation (SD) based on three replicates (*n* = 3). This means that having the same letter(s) within the same column is not significantly different, according to Duncan’s multiple range tests at the 5% probability level.Control (A): ½ MS + 1 mg L^− 1^ riboflavin + 0.1 mgL^− 1^ GA_3_ + 2 g L^− 1^ charcoal), MS = Murashige and Skoog; Ribo = riboflavin; IBA = indole-3-butyric Acid.

### Acclimatization

**Fig. 3:**
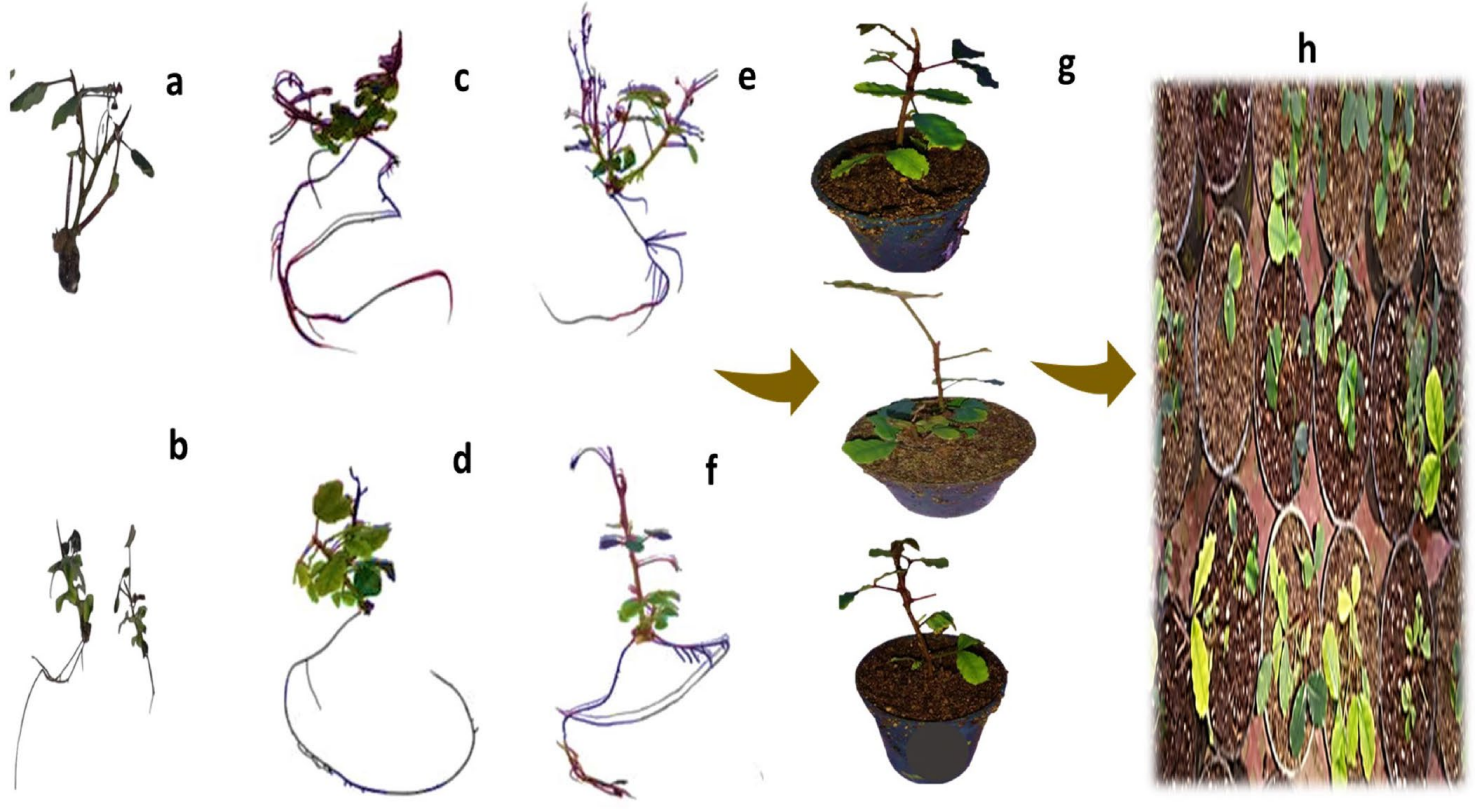
In vitro root behavior and acclimatization stage of Micropropagation in *Ceratonia siliqua* : **(a)** Control (A) ½ MS + 1 mg L^− 1^ riboflavin + 0.1 mg/L GA_3_ + 2 g L^− 1^ charcoal); **(b)** rooting induction in vitro by applying 4 mg L^**− 1**^ IBA; **(c**,** d)** rooting ability by applying 3mg L^**− 1**^ IBA; **(e**,** f)** rooting ability by applying 3 mg L^**− 1**^ IBA + 5 mg L^**− 1**^ Fe_2_O_3_ NPs; **(g)** prepared rooting plantlets for the acclimatization stage; **(h)** acclimatization plants to the greenhouse.

The obtained rooted *Ceratonia siliqua* L. plantlets from in vitro culture media that were added with various levels of Fe_2_O_3_ NPs and IBA were adapted in peat: clay: perlite (1:1:1)(v: v:v). The surviving acclimatized plants were transferred to the greenhouse at the National Research Centre. The success rate for rooting seedlings was 60% (Fig. 3).

### Chemical composition

Chemical analysis was conducted to compare micropropagated shoots with the mother plant of Ceratonia siliqua. Data presented in Table 4 revealed significant variation in secondary metabolites among treatments. The highest total flavonoid content (104.00 µg g⁻¹ FW) was recorded in shootlets cultured on full-strength MS medium supplemented with 1 mg L⁻¹ riboflavin, 1 mg L⁻¹ BA, 0.1 mg L⁻¹ NAA, and 0.1 mg L⁻¹ GA₃. This value showed no significant difference compared with the mother plant (101.92 µg g⁻¹ FW). The lowest flavonoid content (37.00 µg g⁻¹ FW) was observed in shootlets cultured on half-strength MS medium supplemented with 1 mg L⁻¹ riboflavin alone. The mother plant exhibited the highest total phenol and tannin contents (998.89 and 85.20 µg g⁻¹ FW, respectively). Among in vitro treatments, shootlets cultured on half-strength MS medium supplemented with 1 mg L⁻¹ riboflavin and 0.5 mg L⁻¹ BA recorded the highest total phenol and tannin contents (941.16 and 75.84 µg g⁻¹ FW, respectively). The total sugar content was highest in the mother plant (63.15%), followed by shootlets cultured on full-strength MS medium supplemented with 1 mg L⁻¹ riboflavin, 1 mg L⁻¹ BA, 0.1 mg L⁻¹ NAA, and 0.1 mg L⁻¹ GA₃ (57.09%). Comparable values were observed in shootlets cultured on half-strength MS medium supplemented with 1 mg L⁻¹ riboflavin and 1 mg L⁻¹ BA (57.02%) and in full-strength MS medium containing riboflavin, BA, NAA, and GA₃ (50.74%)0.0.1 mg L⁻¹ GA₃ (57.01%).

**Table 4:** Chemical composition of micropropagation treatments and the mother plant of *Ceratonia siliqua* L.

Treatments	Flavonoids )µg g⁻¹ FW(	Total Phenolic )µg g⁻¹ FW(	Tannin )µg g⁻¹ FW(	Total sugar %
Mother plant	101.92 ± 0.02^ab^	998.89 ± 0.30^a^	85.21 ± 0.39^a^	63.15 ± 0.53^a^
½ MS + 1 mg L^− 1^ Ribo	37.00 ± 1.00^h^	360.83 ± 2.50^k^	24.68 ± 0.63^g^	16.53 ± 0.25^i^
½ MS + 1 mg L^− 1^ Ribo + 0.5 mg L^− 1^ BA	95.00 ± 1.00^c^	941.16 ± 0.50^b^	75.84 ± 0.18^b^	24.43 ± 0.63^g^
½ MS + 1 mg L^− 1^ Ribo + 1 mg L^− 1^ BA	63.00 ± 3.00 ^f^	820.83 ± 4.17^d^	57.59 ± 0.63^c^	57.02 ± 0.62^b^
½ MS + 1 mg L^− 1^ Ribo + 0.5 mg L^− 1^ BA + 0.1 mg L^− 1^ NAA + 0.1 mg L^− 1^ GA_3_	99.00 ± 2.64^ab^	811.66 ± 5.00^e^	75.08 ± 0.40^b^	54.50 ± 0.62^c^
½ MS + 1 mg L^− 1^ Ribo + 1 mg L^− 1^ BA + 0.1 mg L^− 1^ NAA + 0.1 mg L^− 1^ GA_3_	59.00 ± 1.00^g^	373.89 ± 1.92^j^	36.70 ± 0.00^f^	23.17 ± 0.62^h^
Full MS + 1 mg L^− 1^ Ribo	92.00 ± 2.00^d^	562.49 ± 4.16^i^	50.16 ± 0.80^e^	43.22 ± 0.62^f^
Full MS + 1 mg L^− 1^ Ribo + 0.5 mg L^− 1^ BA	90.00 ± 2.00^d^	659.99 ± 1.66^h^	56.98 ± 0.02^c^	49.49 ± 0.62^e^
Full MS + 1 mg L^− 1^ Ribo + 1 mg L^− 1^ BA	73.50 ± 1.50^e^	672.50 ± 2.50^g^	53.16 ± 0.02^d^	49.49 ± 0.63^e^
Full MS + 1 mg L^− 1^ Ribo + 0.5 mg L^− 1^ BA + 0.1 mg L^− 1^ NAA + 0.1 mg L^− 1^ GA_3_	100.00 ± 1.00^ab^	700.33 ± 0.00^f^	56.92 ± 1.98^c^	50.74 ± 0.65^d^
Full MS + 1 mg L^− 1^ Ribo + 1 mg L^− 1^ BA + 0.1 mg L^− 1^ NAA + 0.1 mg L^− 1^ GA_3_	104.00 ± 2.00^a^	904.16 ± 4.10^c^	52.53 ± 1.90^d^	57.09 ± 0.79^b^

Data are presented as mean ± standard deviation (SD) based on three replicates (*n* = 3). Means within the same column followed by identical letter (s) are not significantly different, as determined by Duncan’s multiple range test at a significance level of *p ≤* 0.05. MS = Murashige and Skoog medium; Ribo = riboflavin; BA = 6-benzyladenine; GA_3_ = gibberellic Acid; NAA = naphthalene acetic acid.

### Total antioxidant activity

#### Phosphomolybdenum test

The antioxidant potential of *Ceratonia siliqua* extracts was evaluated using the phosphomolybdenum assay. Scavenging activity ranged from 17.73% to 54.43%, while the mother plant recorded a higher value of 67.25% (Table 5). The highest antioxidant activity (54.43%) was observed in shootlets cultured on half-strength MS medium supplemented with 1 mg L⁻¹ riboflavin and 0.5 mg L⁻¹ BA. A similar value (54.03%) was obtained from shootlets cultured on full-strength MS medium containing 1 mg L⁻¹ riboflavin, 1 mg L⁻¹ BA, 0.1 mg L⁻¹ NAA, and 0.1 mg L⁻¹ GA_3_. The lowest activity (17.73%) was observed in shootlets cultured on half-strength MS medium supplemented with riboflavin alone.

**Table 5:** Antioxidant activity of mother plant *C. siliqua* ethanol extract and micropropagation against DPPH and Ammonium molybdate.

Treatments	Total antioxidant %	DPPH%
Mother plant	**67.25 ± 0.22** ^**a**^	66.35 ± 0.05^d^
½ MS + 1 mg L^− 1^ Ribo	17.73 ± 0.80^h^	11.39 ± 0.34^k^
½ MS + 1 mg L^− 1^ Ribo + 0.5 mg L^− 1^ BA	54.43 ± 0.40^b^	79.11 ± 0.41^a^
½ MS + 1 mg L^− 1^ Ribo + 1 mg L^− 1^ BA	46.36 ± 0.40^c^	69.78 ± 1.07^c^
½ MS + 1 mg L^− 1^ Ribo + 0.5 mg L^− 1^ BA + 0.1 mg L^− 1^ NAA + 0.1 mg L^− 1^ GA_3_	45.16 ± 1.39^cd^	62.96 ± 0.20^e^
½ MS + 1 mg L^− 1^ Ribo + 1 mg L^− 1^ BA + 0.1 mg L^− 1^ NAA + 0.1 mg L^− 1^ GA_3_	21.77 ± 1.16^g^	14.73 ± 0.02^j^
Full MS + 1 mg L^− 1^ Ribo	27.28 ± 0.86^f^	22.19 ± 0.30^i^
Full MS + 1 mg L^− 1^ Ribo + 0.5 mg L^− 1^ BA	38.71 ± 0.80^e^	39.20 ± 0.05^h^
Full MS + 1 mg L^− 1^ Ribo + 1 mg L^− 1^ BA	42.33 ± 1.21^d^	49.03 ± 1.71^g^
Full MS + 1 mg L^− 1^ Ribo + 0.5 mg L^− 1^ BA + 0.1 mg L^− 1^ NAA + 0.1 mg L^− 1^ GA_3_	44.33 ± 1.63^cd^	61.55 ± 1.00^f^
Full MS + 1 mg L^− 1^ Ribo + 1 mg L^− 1^ BA + 0.1 mg L^− 1^ NAA + 0.1 mg L^− 1^ GA_3_	54.03 ± 0.80^b^	75.72 ± 0.55^b^

Data are presented as mean ± standard deviation (SD) based on three replicates (*n* = 3). Means within the same column followed by identical letter (s) are not significantly different, as determined by Duncan’s multiple range test at a significance level of *p ≤* 0.05. MS = Murashige and Skoog medium; Ribo = riboflavin; BA = 6-benzyladenine; GA_3_= gibberellic acid; NAA = naphthalene acetic acid.

#### DPPH test

DPPH scavenging activity ranged from 11.39% to 79.11%. The highest activity (79.11%) was recorded in shootlets cultured on half-strength MS medium supplemented with 1 mg L⁻¹ riboflavin and 0.5 mg L⁻¹ BA. The lowest activity (11.39%) was observed in shootlets cultured on half-strength MS medium supplemented with riboflavin alone.

### Identification of Polyphenols (HPLC)

Table 6 shows the polyphenolic compounds identified in ethanol extracts of Ceratonia siliqua mother plants and micropropagated shoots using high-performance liquid chromatography (HPLC). The mother plant contained relatively high levels of pyrocatechol (58.17 mg L⁻¹) and catechin (54.81 mg L⁻¹), followed by caffeic acid (32.40 mg L⁻¹), syringic acid (30.13 mg L⁻¹), chlorogenic acid (6.14 mg L⁻¹), and coumaric acid (2.90 mg L⁻¹). In contrast, micropropagated shoots cultured on half-strength MS medium supplemented with 1 mg L⁻¹ riboflavin and 0.5 mg L⁻¹ BA showed the detection of additional compounds, namely gallic acid, rutin, and vanillin, which were not detected in the mother plant. These results indicate clear differences in the polyphenolic profiles between the mother plant and in vitro–cultured shoots under different growth conditions.

**Table 6:** Identify polyphenols in the mother plant of *Ceratonia siliqua* ethanol extract and the best treatment for total phenol content using HPLC analysis.

Compounds	Conc. (mg L^− 1^ )	
	Mother plant	½ MS + 1 mg L^− 1^ Ribo + 0.5 mg L^− 1^ BA
Gallic acid	0	15.52
Chlorogenic acid	6.14	1.68
Catechin	54.81	6.11
Methyl gallate	0.46	4.15
Coffeic acid	32.4	2.43
Syringic acid	30.13	2.55
Pyro catechol	58.17	ND
Rutin	0	ND
Ellagic acid	3.4	0.66
Coumaric acid	2.9	0.27
Vanillin	0	0.2
Ferulic acid	1.29	ND
Naringenin	9.29	ND
Rosmarinic acid	0.14	0.42
Daidzein	0.18	0.13
Querectin	0.42	0.47
Cinnamic acid	0.03	ND
Kaempferol	0.64	0.39
Hesperetin	1.12	0.36

ND, not detected.

## Discussion

Plant growth regulators, or PGRs, are an essential class of regulatory molecules that impact plant development^37^. Cytokinins are known to promote cell proliferation, cell elongation, and the induction of shoots from the nodal meristem of explants in vitro cultured. 6-Benzyl-aminopurine (BAP) was reported as the most successful cytokinin for inducing shoots since it can accelerate the growth of axillary buds^38,39^.

In the current study, the significantly highest mean of shooting parameters was obtained from a full MS culture medium supplemented with 1 mg L^− 1^ riboflavin, 1 mg L^− 1^ BA, 0.1 NAA, and 0.1mg L^− 1^ GA_3_, as shown in Table 2. These results agreed with Saïdi et al.^40^, who found that the improvement of multiple shoots of *Ceratonia siliqua* L. was noticed when explants were cultured on an MS culture medium containing BAP. Similarly, Radi et al.^41^ revealed that the level of auxin and the balance between the levels of auxin and cytokinin affected the organic differentiation of the carob tree. In addition, Antonopoulou et al.^42^ reported that using vitamin riboflavin in the MS culture medium yielded the highest percentage of shoots on the peach rootstock. Gautam et al.^43^ observed that adding culture media with BA resulted in the maximum shooting percentage in the carnation plant. Furthermore, the optimum medium to propagate *Haloxylon ammodendron* was 0.5 mg L^− 1^ NAA + 0.5 mg L^− 1^ 6-BA enhanced the budding rate for culture establishment^44^. These could be due to the application of exogenous cytokinin stimulators, which increase shoot production and develop the shoot apex^45^. However, numerous studies have demonstrated the significant impact of cytokinins on multiple-shoot stimulation^44^.

Nanoparticles are of great importance in various industries, including agriculture, manufacturing, and healthcare. Iron nanoparticles (Fe_2_O_3_ NPs) are among the most important sources of plant nutrition due to their ability to release a range of pH values^6,46,47^. So, different levels of Fe_2_O_3_ NPs (0.0, 5.0, and 10.0 mg L^− 1^ ) or IBA at various levels (0.0, 2.0, 3.0, and 4.0 mg L^− 1^ ), alone or in combination with Fe_2_O_3_ NPs, were examined to investigate the potential benefits of Fe_2_O_3_ NPs application in improving shoot and root success. Still, there are few reports on this topic. The data in Table 3 indicated that the most significant mean shooting parameter values were obtained from culture media supplemented with 2 mg L^− 1^ IBA and 10 mg L^− 1^ Fe_2_O_3_ oxide NPs. At the same time, significant rooting was observed in culture media supplemented with 3 mg L^− 1^ IBA alone or in combination with 5 mg L^− 1^ Fe_2_O_3_ oxide NPs. The incomplete sentences in the manuscript have been corrected.

In the present study, iron oxide nanoparticles were deliberately applied exclusively during the rooting stage to investigate their specific effects on root induction and subsequent root growth. Previous research has demonstrated that metallic nanoparticles, particularly iron-based nanoparticles, primarily influence root initiation and elongation by modulating auxin balance, micronutrient availability, and reactive oxygen species (ROS) levels in root tissues^48,49^. Consequently, nanoparticle treatments were intentionally excluded from the multiplication stage to avoid confounding effects on shoot proliferation and metabolic responses.

These data are in agreement with El-Ziat et al.^50^. and Khan et al.^51^, who reported that the application of Fe NPs enhanced root and shoot characteristics of *Stivia rebaudiana*, and *Antigonon leptopus* plants. Fe NPs had increased biomass accumulation in *Dracocephalum kotschyi*^52^. In another study, ElMahdy and Elazab^53^. found that applying Fe_2_O_3_ NPs in the culture medium enhanced the growth rate. Also, this improvement of all tested characteristics by application of Fe_2_O_3_ NPs could be a result of the high mobility and reactivity of the particles. NPs that are smaller than 50 nm can physically cross the cell membrane and cell wall and enter the cytoplasm. This may release the amount of chlorophyll and production^54^. In another study, it was shown that applying different nanoparticulates and various concentrations to *Hibiscus syriacus* L. and *Sequoia sempervirens* L. stimulated root formation in vitro^6,9^.

Auxins are well known for their role in root induction. Exogenous application of auxins such as IBA, IAA, and NAA significantly improves rooting in micropropagated plants^55^. IBA has been widely reported as an effective auxin for root induction in apple cultivars and other plant species^56^. Several studies have also emphasized that auxin type and concentration strongly influence root initiation and development^57^.

Shoot proliferation during the multiplication stage was regulated solely by plant growth regulators (BA, NAA, and GA₃) in combination with riboflavin. Riboflavin functions as a key component in cellular redox reactions, respiration, and antioxidant defense as a precursor of flavin cofactors (Flavin mononucleotide (FMN) and flavin adenine dinucleotide (FAD)). Under in vitro conditions, riboflavin has been reported to enhance shoot growth and stimulate the biosynthesis of secondary metabolites by modulating metabolic and oxidative pathways^58,59^. Therefore, biochemical and antioxidant analyses were conducted exclusively during the multiplication stage, as this phase represents a critical window for evaluating metabolic responses and provides a reliable basis for future physiological studies, including those related to calcification.

According to Tables 4 and 5, and 6, the observed fluctuations in phenolic secondary metabolites are in close agreement with the findings of Miguel^60^, who confirmed that these compounds possess high antioxidant potential. Consistently, condensed tannins, total flavonoids, and phenolic compounds were found in significant proportions in carob leaves^61^. Therefore, the formation and accumulation of these secondary metabolites is crucial for enhancing antioxidant activity through the release of tannins, flavonoids, and phenolic compounds, which also contribute to stimulating plant carbohydrate metabolism^51,62^. The differences in polyphenolic compounds between ethanol extracts of the mother plant and shootlets grown on ½ MS medium supplemented with 1 mg L⁻¹ riboflavin and 0.5 mg L⁻¹ BA (Table 6) indicate differential metabolic responses. Ghanemi et al.^63^ reported several polyphenols in carob leaves, including gallic acid, chlorogenic acid, syringic acid, p-coumaric acid, m-coumaric acid, quercetin 3-O-rutinoside, and quercetin. Similarly, Corsi et al.^64^ identified gallic acid, epigallocatechin-3-gallate, and epicatechin-3-gallate in water extracts of *C. siliqua* leaves. Ethanol (80%) extracts of carob leaves were shown to contain 1,6-digalloyl glucose, 1,2,6-trigalloyl glucose, myricetin glucoside, 1,2,3,6-tetragalloyl glucose, myricetin rhamnoside, and syringic acid, as reported by Hsouna et al.^65^.

Similar observations have been reported in *Cichorium intybus L.*, where BAP, IBA, and NAA led to increased phenolic content and inulin levels^22^. Although higher total phenolic content is generally associated with stronger antioxidant activity, antioxidant performance is largely dependent on the qualitative composition of phenolic compounds rather than their total concentration^66^. Different phenolics exhibit markedly different radical-scavenging efficiencies depending on the number and position of hydroxyl groups, degree of conjugation, and overall redox potential. In vitro culture conditions often promote the biosynthesis of low-molecular-weight phenolics with high antioxidant capacity, such as gallic acid and rutin, which were detected in specific micropropagated treatments but were absent or present at lower levels in the mother plant. These compounds are well documented to possess strong hydrogen-donating and electron-transfer abilities, making them highly effective DPPH radical scavengers^67,68^.

Moreover, although condensed tannins contribute substantially to total phenolic content, their large molecular size and limited solubility may reduce their effectiveness in DPPH-based assays compared with smaller, more reactive phenolics^69^. The phosphomolybdenum assay, which measures total antioxidant capacity by reducing Mo(VI) to Mo(V), often yields high activity values in carob extracts due to the strong reducing power of tannins and flavonols, particularly under acidic assay conditions^69^. These methodological differences help explain the apparent discrepancy between total phenolic levels and antioxidant activity observed among the evaluated treatments.

Under controlled in vitro conditions, plant tissues experience unique microenvironmental factors, including altered nutrient availability, exogenous hormones, fixed carbon source, and controlled light intensity that reshape metabolic pathways and modulate phenolic accumulation^70^. In line with this, the present study shows that micropropagation of *Ceratonia siliqua* under specific hormonal regimes (BAP, IBA, NAA) and riboflavin supplementation effectively redirects secondary metabolism toward enhanced phenolic production. This finding is consistent with earlier studies highlighting the role of cytokinins, such as BAP, in regulating phenolic biosynthesis in cultured tissues. For instance, Costa-Pérez et al.^71^ demonstrated that BA combined with UV-C enhanced total flavonoids and hydroxycinnamic acids in *C. siliqua* shoots. Similarly, Sayed et al.^72^. reported that particular light spectra, enhanced phenolic accumulation and antioxidant activity in carob tissues.In another study, it was shown that applying 0.2 mg L^− 1^ BA in *Hibiscus syriacus* L. and *Sequoia sempervirens* L. increased the formation of phenols and flavonoids in vitro^6,9^.

Overall, the present findings confirm that micropropagation of *Ceratonia siliqua* under specific hormonal regimes and riboflavin supplementation not only increases the total phenolic pool but also alters the qualitative phenolic profile. These metabolic changes are attributed to culture-induced physiological modulation rather than genetic variation, highlighting the potential of in vitro culture systems as effective tools for improving the functional and nutraceutical value of carob.

## Conclusions

The application of different growth regulators and riboflavin is an efficient regeneration protocol for the micropropagation of *Ceratonia siliqua L.*, an economically crucial woody tree. The optimized multiplication protocol will serve as an alternative tool for *Ceratonia siliqua* L. large-scale propagation and conservation. The most effective treatment for in vitro culture establishment was full MS supplemented with 1 mg L^− 1^ riboflavin, 1 mg L^− 1^ BA, 0.1 mg L^− 1^ NAA, and 0.1 mg/L GA_3_. Using Iron nanoparticles and IBA, either separately or in combination, promoted the growth of roots and enhanced shoots of *Ceratonia siliqua* L. When comparing the obtained multiplied shoots to the mother plants, it was found that most growth regulator treatments of carob tissue cultured produced high levels of phenolic compound content and antioxidant activity.

## Supplementary Information

Below is the link to the electronic supplementary material.

Supplementary Material 1

Supplementary Material 2

Supplementary Material 3

Supplementary Material 4

Supplementary Material 5

Supplementary Material 6

Supplementary Material 7

Supplementary Material 8

## Data Availability

The datasets used and/or analyzed during the current study are available from the corresponding author on reasonable request.
