# Supplementary material for: Synergistic effects of plant growth regulators, riboflavin, and iron nanoparticles on secondary metabolites in micropropagated Ceratonia siliqua L
Source: Sci Rep. 2026 Jun 4;16:17378. doi: 10.1038/s41598-026-55883-1 (PMC13237283; doi:10.1038/s41598-026-55883-1)
Supplement: Supplementary file 2 — Supplementary Material 2 [file 41598_2026_55883_MOESM2_ESM.doc]

**Table legends(Supplementary part)**

**Table S1.**Shooting ability of different treatments of Carob Tree (*Ceratonia siliqua* L) (Three replicates), treatments as follows as 1: ½ MS+1.0 mg/l riboflavin,2: ½ MS+1.0 mg/l riboflavin +0.5 mg/l BA,3:½MS+1.0 mg/l riboflavin +1.0 mg/lBA,4:½ MS+1.0 mg/l riboflavin +0.5 mg/l BA + 0.1mg/l NAA +0.1 mg/lGA_3_,5:½ MS+1.0 mg/l riboflavin +1.0 mg/lBA + 0.1 mg/l NAA + 0.1 mg/l GA_3_,6:full MS+1.0 mg/l riboflavin,7:full MS+1.0 mg/l riboflavin +0.5 mg/l BA,8:full MS+1.0 mg/l riboflavin +1.0 mg/l BA,9:full MS+1.0 mg/l riboflavin +0.5 mg/l BA + 0.1 mg/l NAA +0.1 mg/l GA3,10:full MS+1.0 mg/l riboflavin +1.0 mg/lBA + 0.1 mg/l NAA + 0.1mg/l GA_3_.

**Table S2**. Shooting and rooting ability of different treatments of Carob Tree (*Ceratonia siliqua* L) (Three replicates), treatments as follows as 1:control (A)**,2:**A + 5.0 mg/l Fe_2_O_3_ NPs**, 3:**A +10.0 mg/l Fe_2_O_3_ NPs**,4:** A + 2.0 mg/l IBA**, 5:** A +2.0 mg/l IBA+5.0 mg/l Fe_2_O_3_ NPs**,6:**A +2.0 mg/l IBA+10.0 mg/l Fe_2_O_3_ NPs**,7:** A +3.0 mg/l IBA**,8:**A +3.0 mg/l IBA+5 mg/l Fe_2_O_3_ NPs**,9:**A +3.0 mg/l IBA+10 mg/l Fe_2_O_3_ NPs**,10:**A +4.0 mg/l IBA**,11:**A +4.0 mg/l IBA+5 mg/l Fe_2_O_3_ NPs**,12:**A +4.0 mg/l IBA+5 mg/l Fe_2_O_3_ NPs, Control (A) → ½ Ms+1 mg/l riboflavin +0.1GA_3_ mg/l + 2 g/l charcoal).

**Table S3**. Flavonoids, Phenol, Tannin, and Total sugar of micropropagation treatments and mother plant of *Ceratonia siliqua* L***.***Treatments as follows as 1: Mother plant, 2:½ MS+1.0 mg/l riboflavin,3: ½ MS+1.0 mg/l riboflavin +0.5 mg/l BA,4:½MS+1.0 mg/l riboflavin +1.0 mg/lBA,5:½ MS+1.0 mg/l riboflavin +0.5 mg/l BA + 0.1mg/l NAA +0.1 mg/lGA_3_,6:½ MS+1.0 mg/l riboflavin +1.0 mg/lBA + 0.1 mg/l NAA + 0.1 mg/l GA_3_,7:full MS+1.0 mg/l riboflavin,8:full MS+1.0 mg/l riboflavin +0.5 mg/l BA,9:full MS+1.0 mg/l riboflavin +1.0 mg/l BA,10:full MS+1.0 mg/l riboflavin +0.5 mg/l BA + 0.1 mg/l NAA +0.1 mg/l GA3,11:full MS+1.0 mg/l riboflavin +1.0 mg/lBA + 0.1 mg/l NAA + 0.1mg/l GA_3_.

**Table S4**. Antioxidant activity of mother plant *C. siliqua* ethanol extract treatments and mother plant of *Ceratonia siliqua* L***.***Treatments follows as 1: Mother plant, 2:½ MS+1.0 mg/l riboflavin,3: ½ MS+1.0 mg/l riboflavin +0.5 mg/l BA,4:½MS+1.0 mg/l riboflavin +1.0 mg/lBA,5:½ MS+1.0 mg/l riboflavin +0.5 mg/l BA + 0.1mg/l NAA +0.1 mg/lGA_3_,6:½ MS+1.0 mg/l riboflavin +1.0 mg/lBA + 0.1 mg/l NAA + 0.1 mg/l GA_3_,7:full MS+1.0 mg/l riboflavin,8:full MS+1.0 mg/l riboflavin +0.5 mg/l BA,9:full MS+1.0 mg/l riboflavin +1.0 mg/l BA,10:full MS+1.0 mg/l riboflavin +0.5 mg/l BA + 0.1 mg/l NAA +0.1 mg/l GA3,11:full MS+1.0 mg/l riboflavin +1.0 mg/lBA + 0.1 mg/l NAA + 0.1mg/l GA_3_.

**Table S5.** Identify polyphenols in the mother plant of *Ceratonia siliqua* ethanol extract and the best treatment for total phenol content using HPLC analysis. sample 1:Mother plant Sample 2S:½ MS+1 mg/l Ribo+0.5 mg/l BA

**ND^a^,** not detected
