# Supplementary material for: Synergistic effects of plant growth regulators, riboflavin, and iron nanoparticles on secondary metabolites in micropropagated Ceratonia siliqua L
Source: Sci Rep. 2026 Jun 4;16:17378. doi: 10.1038/s41598-026-55883-1 (PMC13237283; doi:10.1038/s41598-026-55883-1)

**
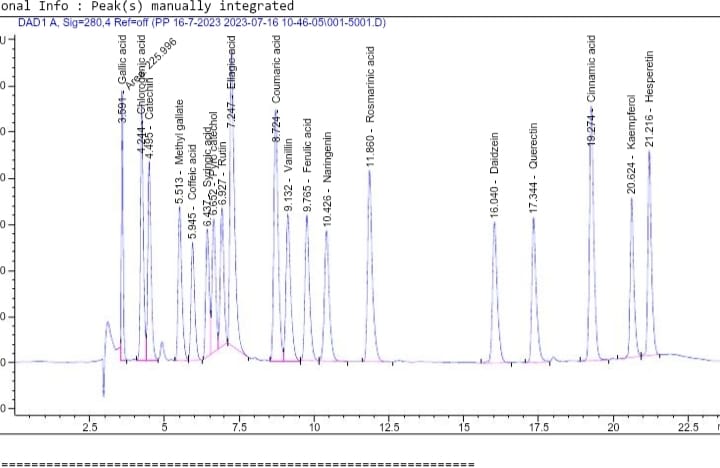
****Fig.S1.** HPLC data chromatography to standard

**Fig.S2.** HPLC data chromatography to sample 1,1:Mother plant


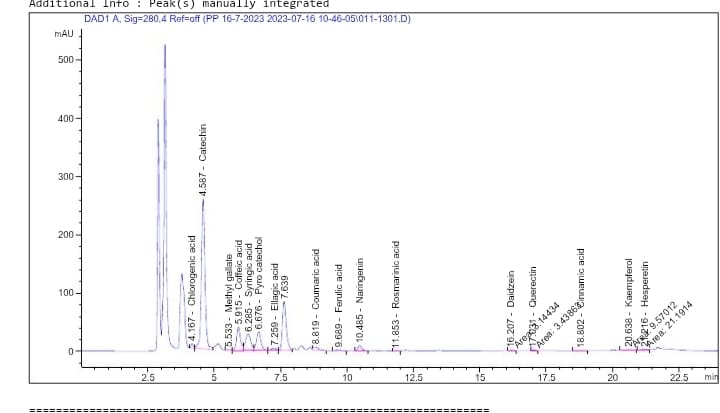


**Fig.S3.** HPLC data chromatography to sample 2, 2**: ½ MS+1 mg/l Ribo+0.5 mg/l BA**


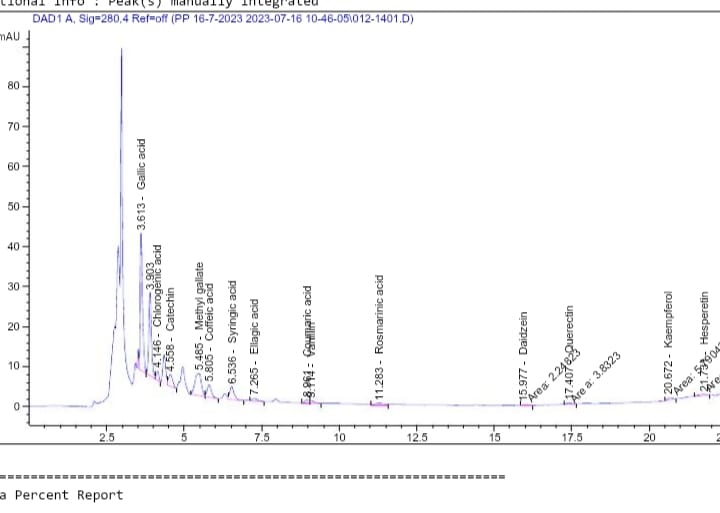

Supplement: Supplementary file 8 — Supplementary Material 8 [file 41598_2026_55883_MOESM8_ESM.docx]
